# Evaluation of the efficacy of chlorous acid water and sodium hypochlorite solution against SARS-CoV-2 in the presence of organic matter

**DOI:** 10.1099/acmi.0.000984.v3

**Published:** 2025-09-19

**Authors:** Basirat Mojisola Lawal-Ayinde, Kosuke Oda, Abeer Mohamed Abdelfattah Elsayed, Tomoyuki Akita, Miuko Kurose, Hiroaki Sasaki, Toshihito Nomura, Akima Yamamoto, Akifumi Higashiura, Isanori Horiuchi, Hisataka Goda, Takemasa Sakaguchi

**Affiliations:** 1 Department of Virology, Graduate School of Biomedical and Health Sciences, Hiroshima University, 1-2-3 Kasumi, Minami-ku, Hiroshima 734-8551, Japan; 2 Department of Pharmacy, Faculty of Pharmacy, Yasuda Women’s University, 6-13-1 Yasuhigashi, Asaminami-ku, Hiroshima 731-0153, Japan; 3 Department of Epidemiology, Infectious Disease Control, and Prevention, Graduate School of Biomedical and Health Sciences, Hiroshima University, 1-2-3 Kasumi, Minami-ku, Hiroshima 734-8551, Japan; 4 Department of Infectious Diseases, Hiroshima University Hospital, 1-2-3 Kasumi, Minami-ku, Hiroshima 734-8551, Japan; 5 Sankei Co., Ltd., 2-2-53 Shiromi, Chuo-ku, Osaka 540-0001, Japan

**Keywords:** COVID-19, free available chlorine (FAC), oxychlorine-based disinfectants, virucidal agents, virus inactivation capability

## Abstract

Chlorous acid water and sodium hypochlorite solution are effective disinfectants against severe acute respiratory syndrome coronavirus-2 (SARS-CoV-2), the virus that caused the pandemic. Recent studies have shown that both compounds have equivalent inactivation effects when tested on purified viruses. However, in practical applications, the presence of organic matter is common and can significantly affect disinfectant performance. We conducted several experiments comparing these two disinfectants under different conditions to better understand their practical efficacy. When an infected cell culture medium (serum-free) was used as the test virus, chlorous acid water and sodium hypochlorite solution showed reduced efficacy. This decrease was attributed to the presence of aa in the medium. Notably, sodium hypochlorite solution showed a more pronounced reduction in potency compared with chlorous acid water. In addition, we evaluated the SARS-CoV-2 inactivation effects of chlorous acid water and sodium hypochlorite solution under various organic loading conditions simulating real-world contamination scenarios such as blood, vomit and saliva. The organic materials used included BSA, SRBCs, polypeptone, FBS and artificial saliva. The results showed that chlorous acid water demonstrated superior resilience to organic matter interference compared with sodium hypochlorite solution. These results suggest that chlorous acid water may be more effective than sodium hypochlorite solution in inactivating viruses on contaminated surfaces, particularly in healthcare settings where organic contamination is common. In summary, our research suggests that chlorous acid water may be a more effective disinfectant in practical settings.

## Data Summary

All data leading to the conclusions drawn in the article are included in the article or supplementary materials.

## Introduction

** **The emergence and global spread of the novel coronavirus [severe acute respiratory syndrome coronavirus-2 (SARS-CoV-2)] have presented an unprecedented public health crisis [1]. SARS-CoV-2 can remain viable on environmental surfaces for extended periods. Aerosolized virus particles have been shown to retain infectivity for up to 3 h on plastic surfaces and up to 72 h on stainless steel surfaces [2]. Notably, the virus exhibits greater stability and infectivity on smooth, non-porous surfaces [3,4]. While human-to-human transmission primarily occurs via respiratory droplets, fomite transmission through contaminated surfaces is also recognized as an important route of infection [5]. These findings underscore the critical importance of effective and safe disinfection strategies, particularly in public spaces and healthcare settings.

In recent years, various chlorine-based oxidizing disinfectants – including chlorine dioxide (ClO₂), sodium hypochlorite solution (NaClO) and chlorous acid water (HClO₂) – have been reported to exert potent inactivating effects against SARS-CoV-2 [6,10]. These agents are believed to exert their microbicidal effects by denaturing viral proteins through intense oxidative action [11,12], with sodium hypochlorite solution being the most widely used among them.

Recent studies employing highly purified SARS-CoV-2 particles have demonstrated that both chlorous acid water (HClO₂) and sodium hypochlorite solution (NaClO) possess comparably high virucidal efficacy [13]. The 99% inactivation concentration (IC₉₉) after 1-min exposure was reported as 0.41 ppm for HClO₂ and 0.54 ppm for NaClO, with no significant difference between the two. However, these studies were conducted under ideal experimental conditions that may not accurately reflect real-world usage scenarios. In actual contamination settings, various organic materials such as blood, saliva and vomitus coexist with the virus. It is well-known that hypochlorite ions, generated from sodium hypochlorite, rapidly lose antimicrobial activity upon contact with organic substances [14]. Similarly, the virucidal activity of chlorous acid water against SARS-CoV-2 has been reported to decrease in the presence of 5% FBS [8]. These observations suggest that organic matter can significantly interfere with the efficacy of chlorine-based disinfectants.

Tests under ideal conditions using purified virus preparations allow us to evaluate the intrinsic activity of disinfectants. However, in real-world settings, the presence of diverse organic contaminants can affect disinfectant performance, and these interactions have not been sufficiently elucidated. In particular, there is a lack of systematic investigation into how different types of organic load influence each disinfectant and how their relative efficacy may change under such conditions.

This study aims to address these knowledge gaps through the following novel approaches. (1) Systematic evaluation under various organic load conditions: We established experimental models simulating real-world contamination scenarios, including blood contamination [BSA and sheep red blood cells (SRBCs)], vomitus contamination [polypeptone (PP)] and saliva contamination (FBS and artificial saliva), and quantitatively compared disinfectant efficacy under each condition. (2) Establishment of a practical evaluation system: We employed virus-containing culture supernatants from infected cells, which include media components (e.g. aa) that cannot be accounted for in purified virus systems, to better reflect practical application environments. (3) Quantitative comparison of relative disinfectant efficacy: Using the Chick–Watson model, we numerically analysed the changes in virucidal potency of chlorous acid water and sodium hypochlorite solution under varying organic load conditions and compared them from a practical usability perspective.

The findings of this study are expected to contribute directly to the development of effective disinfection protocols in healthcare and public facility settings and to provide a scientific basis for infection control strategies. In particular, this research may inform the selection of more effective disinfectants in healthcare environments, where organic contamination is commonplace.

## Methods

### Regents

Chlorous acid water (Klorus Acid・N barrier) was provided by Sankei Co., Ltd. (Osaka, Japan), and sodium hypochlorite solution (PURELOX-S) was purchased from Oyarox Co., Ltd. (Osaka, Japan). Sodium thiosulphate and PP (HIPOLYPEPTON-N) were purchased from FUJIFILM Wako Pure Chemical Corporation (Tokyo, Japan). BSA was purchased from Katayama Chemical Industries Co. (Osaka, Japan). SRBCs were purchased from JAPAN BIO SERUM CO. (Tokyo, Japan). FBS was purchased from BIOSERA (Kansas City, MO, USA). Artificial saliva ASTM E2720-16/ASTM E2721-16 with mucin (catalogue #1700–0316) was purchased from Pickering Laboratories (Mountain View, CA, USA).

The free available chlorine (FAC) concentrations of chlorous acid water and sodium hypochlorite solution were measured immediately before the experiment using the DPD method with an RC-V2-CAW meter (Kasahara Rika Kogyo, Saitama, Japan) [15]. These solutions were diluted with sterile distilled water, and the FAC values of the diluted solutions were also confirmed. Based on the FAC values, comparisons were made between chlorous acid water and sodium hypochlorite solution.

### Cells and virus

VeroE6/TMPRSS2 cells (African green monkey kidney cells expressing human TMPRSS2) were provided by the Japanese Collection of Research Bioresources (JCRB) Cell Bank (JCRB1819) [16]. The SARS-CoV-2/JP/Hiroshima-46059T/2020 strain (B.1.1, GISAID accession ID: EPI_ISL_6289932) was inoculated into VeroE6/TMPRSS2 cells and cultured in Dulbecco’s modified Eagle’s medium (DMEM; FUJIFILM Wako Pure Chemical Corporation). Infected cell culture fluid was collected 48 h after infection, clarified by low-speed centrifugation and filtered through a 0.45-µm filter to obtain the test virus. Virus titres were measured using the standard 50% tissue culture infectious dose (TCID_50_) method and expressed as TCID_50_/ml [17]. All experiments handling infectious SARS-CoV-2 were conducted in the biosafety level 3 facility of Hiroshima University.

### Antiviral test and modelling

In the antiviral tests, chlorous acid water and sodium hypochlorite solution were placed in polystyrene tubes, and the test reagents were prepared for incubation with the virus. Test reagents (each 90 µl) were reacted with the virus (10 µl) at room temperature for 10 min, and the reaction mixture was diluted tenfold in DMEM containing 10% FBS and 10 µM sodium thiosulphate to stop the reaction of the disinfectant. This mixture was serially diluted tenfold in 10% FBS-DMEM and inoculated into monolayers of VeroE6/TMPRSS2 cells in 96-well plates. Inoculated cells were cultured in a CO_2_ incubator until the cytopathic effect (CPE) was fully expressed. The CPE was observed under a microscope, and TCID_50_/ml values were calculated using the Behrens–Kärber method [18]. Experimental results demonstrated that sodium thiosulphate addition does not affect viral replication or infectivity titre measurements (Fig. S1, available in the online Supplementary Material).

The following reagents were used in protein load experiments. For experiments using a final concentration of 0.5% PP, a 10% (w/v) PP aqueous solution was prepared, sterilized by filtration through a 0.1-µm filter and mixed with an equal volume of the virus. The PP concentration in the mixture was 5% (w/v), and the final PP concentration in the reaction water was 0.5% as the virus and mixture were mixed in a 1 : 9 ratio. For tests using 0.03% BSA, a 0.6% (w/v) BSA aqueous solution was similarly prepared, and the final concentration in the reaction water was 0.03%. For the 5% (v/v) FBS test, 100% FBS was used to achieve a final concentration of 5%.

In experiments with a final concentration of [0.3% SRBCs+0.3% BSA], the virus, [3% SRBCs+3% BSA] and disinfectant were mixed in a ratio of 1 : 1 : 8. In experiments with a final concentration of [artificial saliva], the virus, artificial saliva and disinfectant were mixed in a ratio of 1 : 1 : 8. In this case, a 1.25-fold-concentrated disinfectant was used to adjust the final concentration to 1×.

Experimental data were fitted to the Chick–Watson model [19].


$$Log_{10}\left[\frac{N\left(t\right)}{N_{0}}\right]=-k\cdot C^{n}\cdot t$$

In equation 1, *N*(*t)* is the remaining virus infectivity after incubation with the disinfectant, and *N*_0_ is the initial infectivity. *C* is the given disinfectant concentration, and *t* is the incubation time (minutes) with the disinfectant. *k* and *n* are the rate constant and the dilution coefficient that determine the relative importance of the disinfectant concentration for each disinfectant, respectively, and were estimated by the nonlinear least-squares method. The 95% CIs for the fitted curves of the Chick–Watson model were calculated using JMP (SAS Institute, Cary, NC, USA).

## Results

### Effects of chlorous acid water and sodium hypochlorite solution on SARS-CoV-2

**Fig. 1.**
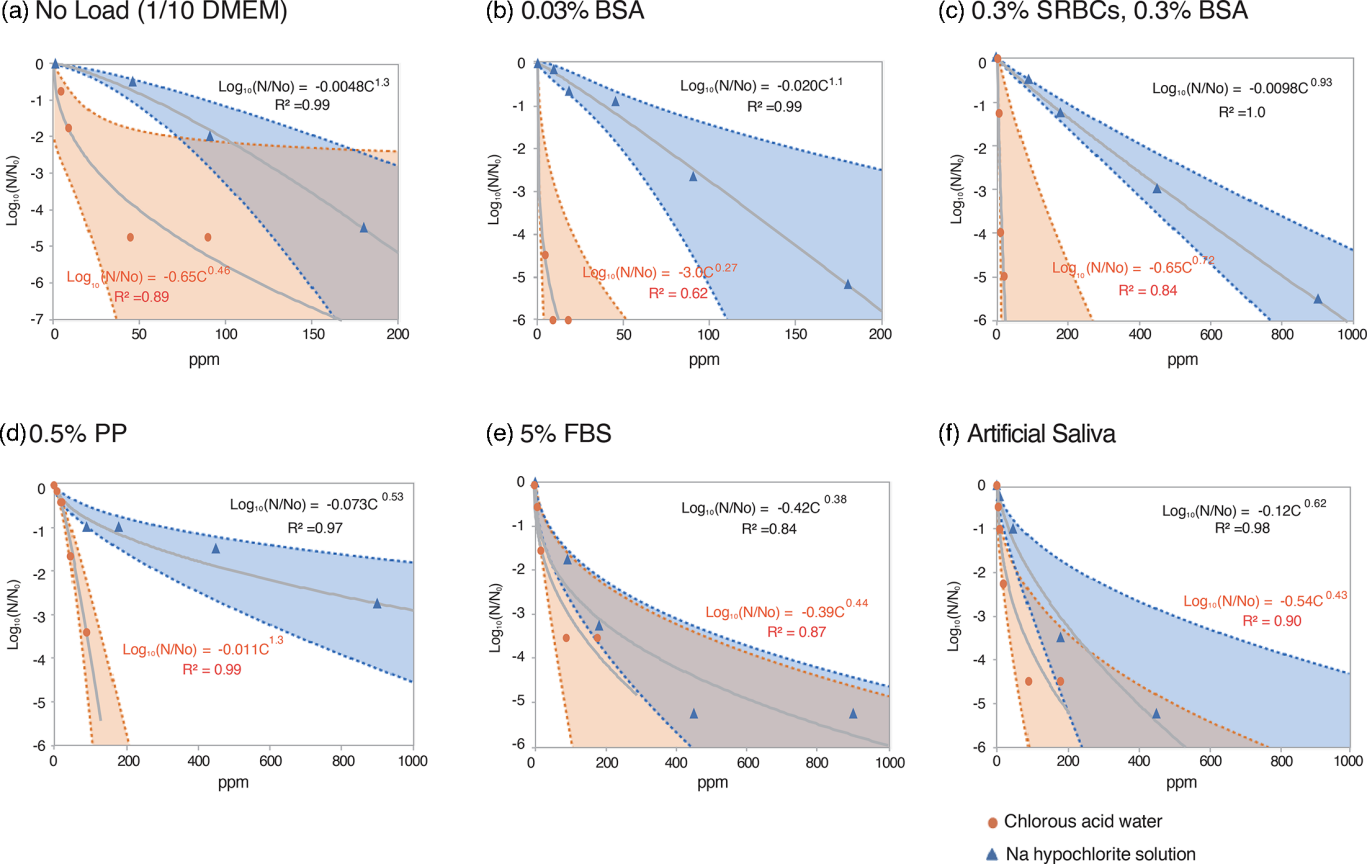
Inactivation of SARS-CoV-2 by chlorous acid water and sodium hypochlorite solution in the presence of organic matter. SARS-CoV-2 was incubated with oxychlorine disinfectants in the presence of 0.03% BSA (**b**), [0.3% SRBCs+0.3% BSA] (**c**), 0.5% PP (**d**), 5% FBS (**e**), artificial saliva (**f**) or no organic matter (containing 1/10-diluted DMEM) (**a**) for 10 min, and infectivity was measured by the TCID₅₀ method. Data points are shown as red circles (chlorous acid) and blue triangles (sodium hypochlorite solution). Measured values were fitted to an approximate equation based on the Chick–Watson model. Approximation equations and *R*² values are shown in the graphs. *R*², coefficient of determination; Log₁₀(N/N₀), log reduction in survival ratio. The 95% CIs for the fitted curves were calculated using JMP and are coloured in red (chlorous acid) and blue (sodium hypochlorite solution).

The culture supernatant of infected cells (serum-free) was exposed to chlorous acid water or sodium hypochlorite solution for 10 min, and the relationship between FAC and infectivity was analysed using the Chick–Watson model (Fig. 1a and Table S1). The 50%, 99%, 99.9% and 99.99% inhibitory concentrations (IC₅₀, IC₉₉, IC₉₉.₉ and IC₉₉.₉₉) of both solutions were calculated from these fitted curves (Table S2). The IC₅₀ of chlorous acid water was 0.19 ppm, IC₉₉ was 11 ppm, IC₉₉.₉ was 27 ppm, and IC₉₉.₉₉ was 50 ppm. For sodium hypochlorite solution, these values were 23, 97, 132 and 164 ppm, respectively (Table S2).

Subsequent comparisons were made carefully, primarily based on IC₉₉, because the IC₅₀ values showed significant variability. This variability may be attributed to the calculation method, which was designed to account for a reduction in viral titre from 10⁷ to the detection limit of 6.3×10¹. Thus, the IC₅₀ value representing a 50% reduction at the end of this range may not be accurately determined.

Previous studies have shown that highly purified SARS-CoV-2 particles with minimal impurities are inactivated by both chlorous acid water and sodium hypochlorite solution. The IC₉₉ values for a 1-min reaction were 0.41 and 0.54 ppm, respectively, with no significant difference between the two solutions [13].

In contrast, in the present study, in which the virus was in the culture supernatant of infected cells, the IC₉₉ value for chlorous acid water was 11 ppm (10-min reaction) (Tables 1 and S2), whereas that for sodium hypochlorite solution was 97 ppm (10-min reaction) (Tables 1 and S2), indicating that sodium hypochlorite solution was approximately nine times less effective. However, the overlapping 95% CIs indicated that the effects of chlorous acid water and sodium hypochlorite solution were similar (Fig. 1a). In this reaction system, the virus solution and disinfectant were mixed in a 1 : 9 ratio, and the infected cell culture supernatant (serum-free DMEM) was diluted tenfold with the disinfectant. The components of the tenfold-diluted infected cell culture supernatant alone significantly reduced the virus-inactivating ability of both disinfectants, possibly due to the influence of aa present in DMEM.

**Table 1.** Comparison of 99% and 99.9% inhibitory concentrations under the condition of organic matter loading

	99% inhibitory concentration: IC_99_ (ppm)			99.9% inhibitory concentration: IC_99.9_ (ppm)		
Reagent	Chlorous acid water (ppm)	Sodium hypochlorite solution (ppm)	Ratio (sodium hypochlorite solution/chlorous acid water)	Chlorous acid water (ppm)	Sodium hypochlorite solution (ppm)	Ratio (sodium hypochlorite solution/chlorous acid water)
No load (1/10 DMEM)	11	97	8.8	27	132	4.9
0.03% BSA	0.22	74	336	0.98	108	110.2
[0.3% SRBCs+0.3% BSA]	4.7	302	64	8.3	466	56.1
0.5% PP	59	499	8.5	81	1,067	13.2
5% FBS	41	58	1.4	101	166	1.6
Artificial saliva	21	91	4.3	54	175	3.2

The table shows the concentrations at which SARS-CoV-2 is inactivated by 99% and 99.9% by chlorous acid water or sodium hypochlorite solution in the presence of various types of organic matter. Also shown are the ratios of IC_99_ and IC_99.9_ values for sodium hypochlorite solution and chlorous water, respectively. Samples labelled ‘no load’ contained DMEM diluted to 1/10 in the reaction. All other organic loading samples also contained DMEM diluted to 1/10.

### Impact of organic load on the efficacy of the disinfectants

Furthermore, the differences in the efficacy of the two disinfectants under different organic load conditions were investigated. To simulate blood contamination, [0.3% SRBCs+0.3% BSA] or 0.03% BSA was used [20]. The virus suspended in cell culture medium was mixed with each organic material and then exposed to chlorous acid water or sodium hypochlorite solution. The reaction was stopped 10 min after initiation, and the infectivity of the remaining virus was measured. Approximation curves were plotted (Fig. 1b, c), and IC₅₀, IC₉₉, IC₉₉.₉ and IC₉₉.₉₉ values were determined (Table S2).

With the addition of 0.03% BSA and [0.3% SRBCs+0.3% BSA], the IC₉₉ values of chlorous acid water decreased from 11 ppm (without additives) to 0.22 and 4.7 ppm, respectively, indicating enhanced viral inactivation (Fig. 1b, c, Tables 1 and S2). The viral inactivation ability of chlorous acid water, which was reduced by amino acids, was restored by the addition of these organic materials. However, the detailed mechanism remains to be elucidated.

In contrast, with the addition of 0.03% BSA and [0.3% SRBCs+0.3% BSA], the IC₉₉ values of sodium hypochlorite solution changed from 97 ppm (without additives) to 74 and 302 ppm, respectively, showing slight decreases and increases, resulting in either similar or further reduced viral inactivation ability (Fig. 1b, c, Tables 1 and S2).

When comparing the effects of 0.03% BSA and [0.3% SRBCs+0.3% BSA] on chlorous acid water and sodium hypochlorite solutions using the IC₉₉ ratio, the value increased significantly from 8.8 (without additives) to 336 (with 0.03% BSA) and 64 (with [0.3% SRBCs+0.3% BSA]). The absence of overlap in the 95% CIs between the two disinfectants indicated a statistically significant difference between them (Fig. 1b, c).

Next, 0.5% PP was used as an organic load to simulate vomit contamination [21]. In the presence of 0.5% PP, the IC₉₉ value of chlorous acid water increased from 11 ppm (without additives) to 59 ppm. For the sodium hypochlorite solution, it increased from 97 ppm (without additives) to 499 ppm, indicating a significant reduction in viral inactivation ability (Fig. 1d, Tables 1 and S2). The 95% CIs also showed no overlap (Fig. 1d), indicating a statistically significant difference in antiviral effects between the two disinfectants.

In addition, 5% FBS [8,13] and artificial saliva [22] were used to simulate saliva contamination. With the addition of 5% FBS and artificial saliva, the IC₉₉ values of chlorous acid water increased from 11 ppm (without additives) to 41 and 21 ppm, respectively, suggesting reduced efficacy of chlorous acid water (Fig. 1e, Tables 1 and S2). For sodium hypochlorite solution, IC₉₉ values decreased slightly to 58 and 91 ppm, respectively, compared to 97 ppm without additives. The IC₉₉.₉ values increased slightly from 132 ppm without additives to 166 and 175 ppm, respectively (Fig. 1f, Tables 1 and S2). This indicates that sodium hypochlorite solution maintained almost the same viral inactivation ability even after the addition of 5% FBS or artificial saliva compared to the condition without additives (including 1/10 diluted DMEM). Since chlorous acid water was significantly influenced by 5% FBS and artificial saliva, the efficacy of the two disinfectants became comparable, with considerable overlap observed in their 95% CIs (Table 1, Fig. 1e, f).

## Discussion

This study demonstrated that the virucidal efficacy of chlorous acid water and sodium hypochlorite solution against SARS-CoV-2 is significantly affected by the presence of organic load. To understand this phenomenon, it is essential to examine the distinct molecular mechanisms of action underlying each disinfectant.

### Fundamental differences in mechanisms of action

Sodium hypochlorite solution exists in aqueous equilibrium between hypochlorous acid (HClO) and hypochlorite ion (ClO⁻), with HClO serving as the primary antimicrobial agent. HClO reacts non-selectively with nucleophilic aa residues – such as cysteine, methionine and tryptophan – found in viral capsid and envelope proteins [11]. While highly reactive, hypochlorous acid lacks target specificity and readily reacts with various organic substances in the environment.

In contrast, chlorous acid water primarily contains chlorite ion (ClO₂⁻), which exists as chlorous acid (HClO₂) under appropriate pH conditions. The oxidation mechanism of chlorous acid is fundamentally distinct from that of hypochlorous acid, characterized by selective one-electron transfer reactions [12]. This selectivity allows chlorous acid to preferentially oxidize specific functional groups – particularly phenolic hydroxyls and indole rings – while minimizing reactions with nonspecific organic matter.

### Differential responses to organic load and their mechanisms

The reduction in disinfectant efficacy due to aa present in the culture supernatant can be explained by different mechanisms for each agent. Hypochlorous acid reacts indiscriminately with all aa, resulting in substantial depletion of active chlorine by free aa in the medium. In particular, reactive aa – such as cysteine, methionine and histidine – undergo rapid reaction with hypochlorous acid, leading to a sharp decline in antimicrobial potency.

In contrast, chlorous acid exhibits selective reactivity towards aa, with detectable interaction limited to a few residues (e.g. histidine and cysteine) [23]. This selectivity greatly reduces the consumption of active species by medium components, as compared with sodium hypochlorite.

Interestingly, the addition of BSA led to a recovery in the virucidal efficacy of chlorous acid water. This phenomenon may be associated with conformational changes in protein structure induced by partial oxidation, exposing viral binding sites and enhancing access. As a globular protein, BSA may also function as a stabilizer of chlorous acid, prolonging the persistence of its active species. In contrast, no such efficacy recovery was observed for sodium hypochlorite, whose virucidal activity remained diminished even in the presence of BSA – likely due to irreversible consumption of hypochlorous acid through rapid reactions with surface aa residues of BSA.

### Superior resilience to organic load via selective oxidation

The superior resilience of chlorous acid water to organic load, compared with sodium hypochlorite, fundamentally derives from its selective oxidation mechanism. SARS-CoV-2 spike and nucleocapsid proteins are stabilized by intra- and intermolecular disulphide bonds involving multiple cysteine residues. Chlorous acid selectively targets these disulphide linkages, thereby efficiently disrupting the virus’s structural integrity. This target specificity minimizes ‘wasteful’ reactions with surrounding organic matter, preserving antiviral potency.

Within complex organic matrices, chlorous acid water demonstrates greater chemical stability than sodium hypochlorite solution. This enhanced stability can be attributed to (i) suppression of competitive reactions with non-specific organic compounds, (ii) gradual consumption of oxidative potential via stepwise one-electron transfers and (iii) favourable diffusion properties due to moderate molecular size and polarity. These characteristics enable chlorous acid water to maintain its virucidal efficacy even in the presence of substantial organic load.

### Broad-spectrum utility and practical significance

Chlorous acid water has demonstrated superior tolerance to organic matter when inactivating other pathogens, including human norovirus, feline calicivirus and *Clostridium difficile* spores [23,24]. Taken together with the present study’s detailed investigation of SARS-CoV-2 inactivation under varying organic load conditions, the protein tolerance of chlorous acid water is likely applicable across a broad range of pathogens.

The robust resilience of chlorous acid water to organic contaminants, as revealed in this study, holds important implications for its practical use in healthcare settings. In environments where high concentrations of organic matter – such as blood, vomitus and saliva – are frequently encountered, the efficacy of sodium hypochlorite is markedly diminished. In contrast, chlorous acid water retains consistent virucidal activity. This allows for reliable disinfection performance without necessitating the use of excessively high concentrations to compensate for efficacy loss, thereby reducing the risk of occupational exposure for personnel.

### Future directions and perspectives

While the superior organic load resilience of chlorous acid water has been elucidated in this study, several issues remain to be addressed. First, further clarification is needed regarding the precise molecular sites and reaction pathways between chlorous acid and viral proteins. Second, the efficacy of chlorous acid water under real-world conditions of organic contamination in clinical and public settings must be validated. Third, optimization of disinfection parameters – including pH, temperature and contact time – may further enhance its virucidal efficacy. Addressing these challenges will increase the practical utility of chlorous acid water and contribute to the establishment of more effective infection control strategies.

## Supplementary material

10.1099/acmi.0.000984.v3 Uncited Supplementary Material 1.

## Abbreviations

CPE: cytopathic effect
DMEM: Dulbecco's modified Eagle’s medium
FAC: free available chlorine
IC₉₉: 99% inactivation concentration
JCRB: Japanese Collection of Research Bioresources
PP: polypeptone
SARS-CoV-2: severe acute respiratory syndrome coronavirus-2
SRBC: sheep red blood cell
TCID_50_: 50% tissue culture infectious dose

## References

[1] World Health Organization Coronavirus disease (COVID-19) pandemic. https://www.who.int/emergencies/diseases/novel-coronavirus-2019.

[2] van Doremalen N, Bushmaker T, Morris DH, Holbrook MG, Gamble A (2020). Aerosol and surface stability of SARS-CoV-2 as compared with SARS-CoV-1. N Engl J Med.

[3] Chin AWH, Chu JTS, Perera MRA, Hui KPY, Yen H-L (2020). Stability of SARS-CoV-2 in different environmental conditions. *Lancet Microbe*.

[4] Riddell S, Goldie S, Hill A, Eagles D, Drew TW (2020). The effect of temperature on persistence of SARS-CoV-2 on common surfaces. Virol J.

[5] Kampf G, Todt D, Pfaender S, Steinmann E (2020). Persistence of coronaviruses on inanimate surfaces and their inactivation with biocidal agents. J Hosp Infect.

[6] Cimolai N (2022). Disinfection and decontamination in the context of SARS-CoV-2-specific data. J Med Virol.

[7] Greaves J, Fischer RJ, Shaffer M, Bivins A, Holbrook MG (2022). Sodium hypochlorite disinfection of SARS-CoV-2 spiked in water and municipal wastewater. Sci Total Environ.

[8] Hatanaka N, Awasthi SP, Xu B, Goda H, Kawata H (2022). Comparative evaluation of chlorous acid and sodium hypochlorite activity against SARS-CoV-2. *Access Microbiol*.

[9] Hatanaka N, Xu B, Yasugi M, Morino H, Tagishi H (2021). Chlorine dioxide is a more potent antiviral agent against SARS-CoV-2 than sodium hypochlorite. J Hosp Infect.

[10] Hatanaka N, Yasugi M, Sato T, Mukamoto M, Yamasaki S (2022). Hypochlorous acid solution is a potent antiviral agent against SARS-CoV-2. J Appl Microbiol.

[11] Fukuzaki S (2006). Mechanisms of actions of sodium hypochlorite in cleaning and disinfection processes. Biocontrol Sci.

[12] Ogata N (2007). Denaturation of protein by chlorine dioxide: oxidative modification of tryptophan and tyrosine residues. Biochemistry.

[13] Lawal-Ayinde BM, Morita T, Oda K, Nazmul T, Kurose M (2023). Virus purification highlights the high susceptibility of SARS-CoV-2 to a chlorine-based disinfectant, chlorous acid. PLoS One.

[14] Urushidani M, Kawayoshi A, Kotaki T, Saeki K, Mori Y (2022). Inactivation of SARS-CoV-2 and influenza A virus by dry fogging hypochlorous acid solution and hydrogen peroxide solution. PLoS One.

[15] Yamaoka H, Nakayama-Imaohji H, Horiuchi I, Yamasaki H, Nagao T (2016). Tetramethylbenzidine method for monitoring the free available chlorine and microbicidal activity of chlorite-based sanitizers under organic-matter-rich environments. Lett Appl Microbiol.

[16] Matsuyama S, Nao N, Shirato K, Kawase M, Saito S (2020). Enhanced isolation of SARS-CoV-2 by TMPRSS2-expressing cells. Proc Natl Acad Sci USA.

[17] Nomura T, Nazmul T, Yoshimoto R, Higashiura A, Oda K (2021). Ethanol susceptibility of SARS-CoV-2 and other enveloped viruses. Biocontrol Sci.

[18] Nazmul T, Lawal-Ayinde BM, Morita T, Yoshimoto R, Higashiura A (2023). Capture and neutralization of SARS-CoV-2 and influenza virus by algae-derived lectins with high-mannose and core fucose specificities. Microbiol Immunol.

[19] Peleg M (2021). Modeling the dynamic kinetics of microbial disinfection with dissipating chemical agents-a theoretical investigation. Appl Microbiol Biotechnol.

[20] EN13727 (2012). Chemical disinfectants and antiseptics. Quantitative suspension test for the evaluation of bactericidal activity in the medical area. Test method and requirements (phase 2, step 1).

[21] Igimi S, Noda M, Uema M (2017). Research report of inactivation conditions of noroviruses. http://www.mhlw.go.jp/file/06-Seisakujouhou-11130500-Shokuhinanzenbu/0000125854.pdf.

[22] ASTM2721-16 (2023). Standard practice for evaluation of effectiveness of decontamination procedures for surfaces when challenged with droplets containing human pathogenic viruses.

[23] Goda H, Nakayama-Imaohji H, Yamaoka H, Tada A, Nagao T (2022). Inactivation of human norovirus by chlorous acid water, a novel chlorine-based disinfectant. J Infect Chemother.

[24] Goda H, Yamaoka H, Nakayama-Imaohji H, Kawata H, Horiuchi I (2017). Microbicidal effects of weakly acidified chlorous acid water against feline calicivirus and *Clostridium difficile* spores under protein-rich conditions. PLoS One.

