# Supplementary material for: Evaluation of the efficacy of chlorous acid water and sodium hypochlorite solution against SARS-CoV-2 in the presence of organic matter
Source: Access Microbiol. 2025 Sep 19;7(9):000984.v3. doi: 10.1099/acmi.0.000984.v3 (PMC12451309; doi:10.1099/acmi.0.000984.v3)
Supplement: Uncited Supplementary Material 1. [file acmi-7-00984-s001.pdf]

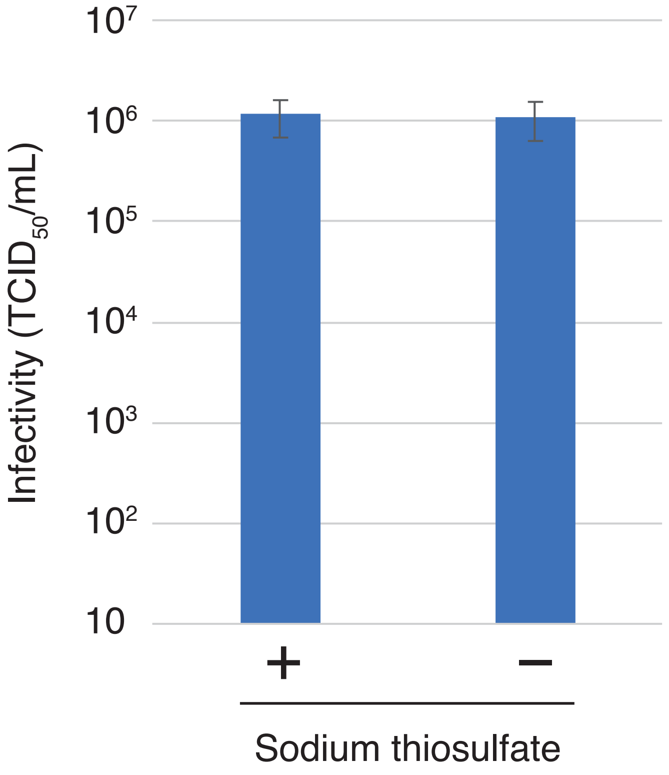

Supplementary Fig. 1. Effect of sodium thiosulfate addition on viral infection

In antiviral assays, experiments were conducted with and without the addition of sodium thiosulfate as a stop solution in the absence of disinfectant to evaluate the effect of the stop solution on viral infectivity. Sterile water was added instead of disinfectant, and 50 μL of this was added to either 450 μL of stop solution (10 μM sodium thiosulfate in DMEM containing 10% FBS) or DMEM containing 10% FBS alone, followed by 10-fold serial dilution to measure viral infectivity titers. Experiments were performed in triplicate, and the mean infectivity titers are shown graphically. Error bars represent standard error.

Supplementary Table 1. Parameters based on the Chick-Watson model

Reagent	k	n	R^2
Chlorous acid water	0.065	0.46	0.89
Chlorous acid water + 0.5% PP	0.0011	1.3	0.99
Chlorous acid water + 0.03% BSA	0.30	0.27	0.62
Chlorous acid water + [0.3% SRBCs + 0.3% BSA]	0.065	0.72	0.84
Chlorous acid water + 5% FBS	0.039	0.44	0.87
Chlorous acid water + Artificial Saliva	0.054	0.43	0.90
Sodium hypochlorite solution	0.00048	1.3	0.99
Sodium hypochlorite solution + 0.5% PP	0.0073	0.53	0.97
Sodium hypochlorite solution + 0.03% BSA	0.0020	1.1	0.99
Sodium hypochlorite solution + [0.3% SRBCs + 0.3% BSA]	0.00098	0.93	1.0
Sodium hypochlorite solution + 5% FBS	0.042	0.38	0.84
Sodium hypochlorite solution + Artificial Saliva	0.012	0.62	0.98

The reagent concentration and viral growth inhibition results were fitted to the Chick-Watson model to calculate the rate constant (k) and dilution coefficient (n). Coefficient of determination (R^2) values were also calculated. The reaction time was 10 minutes.

Supplementary Table 2. Inhibitory concentrations of SARS-CoV-2 with chlorous acid water and sodium hypochlorite solution under organic material loading

	Inhibitory Concentration (ppm)			
Reagent	50% Inhibition	99% Inhibition	99.9% Inhibition	99.99% Inhibition
Chlorous acid water	0.19	11	27	50
Chlorous acid water + 0.03% BSA	0.0002	0.22	0.98	2.8
Chlorous acid water + [0.3% SRBCs, 0.3% BSA]	0.35	4.7	8.3	12
Chlorous acid water + 0.5% PP	13	59	81	101
Chlorous acid water + 5% FBS	0.57	41	101	193
Chlorous acid water + Artificial Saliva	0.25	21	54	107
Sodium hypochlorite solution	23	97	132	164
Sodium hypochlorite solution + 0.03% BSA	13	74	108	142
Sodium hypochlorite solution + [0.3% SRBCs + 0.3% BSA]	39	302	466	635
Sodium hypochlorite solution + 0.5% PP	14	499	1067	1830
Sodium hypochlorite solution + 5% FBS	0.41	58	166	352
Sodium hypochlorite solution + Artificial Saliva	4.4	91	175	277

Based on the Chick-Watson model, as shown in Fig. 1, we calculated the concentrations that would inactivate 50%, 99%, 99.9%, or 99.99% of SARS-CoV-2 when the virus was reacted with chlorous acid water or sodium hypochlorite solution with or without organic matter loading for 10 min. BSA: bovine serum albumin, SRBCs: sheep red blood cells, PP: polypeptone, FBS: fetal bovine serum.
